# Supplementary figures and images for: Glucocorticoid signaling in pancreatic islets modulates gene regulatory programs and genetic risk of type 2 diabetes
Source: PLoS Genet. 2021 May 13;17(5):e1009531. doi: 10.1371/journal.pgen.1009531 (PMC8183998; doi:10.1371/journal.pgen.1009531)

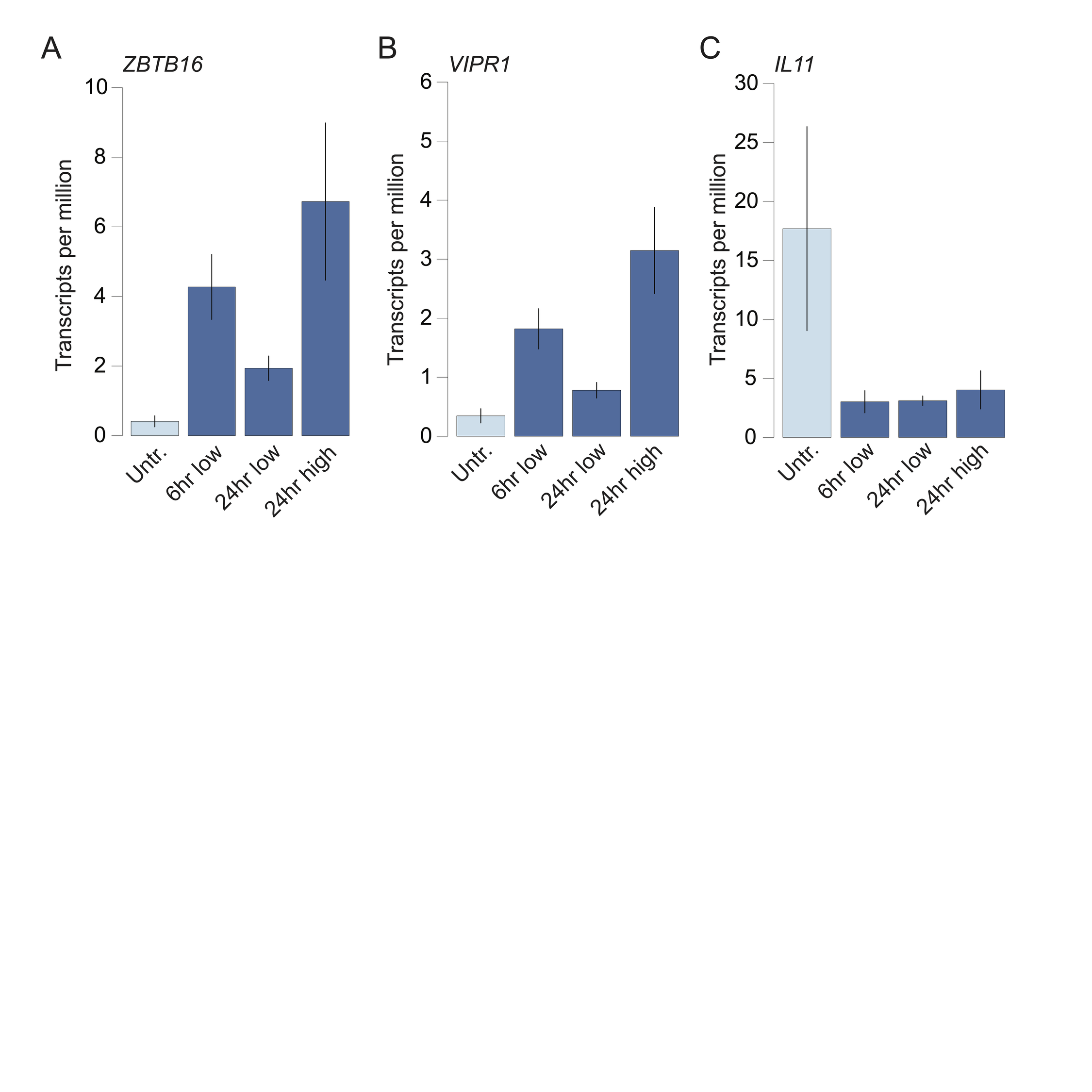

Supplement: S1 Fig — Expression level of (A) ZBTB16, (B) VIPR1 and (C) IL11 in high-dose (100ng/mL for 24hr), low-dose (4ng/mL for 24hr or 6hr) glucocorticoid-treated or untreated islets. Values represent mean expression and standard error. (TIF) [file pgen.1009531.s001.tif]

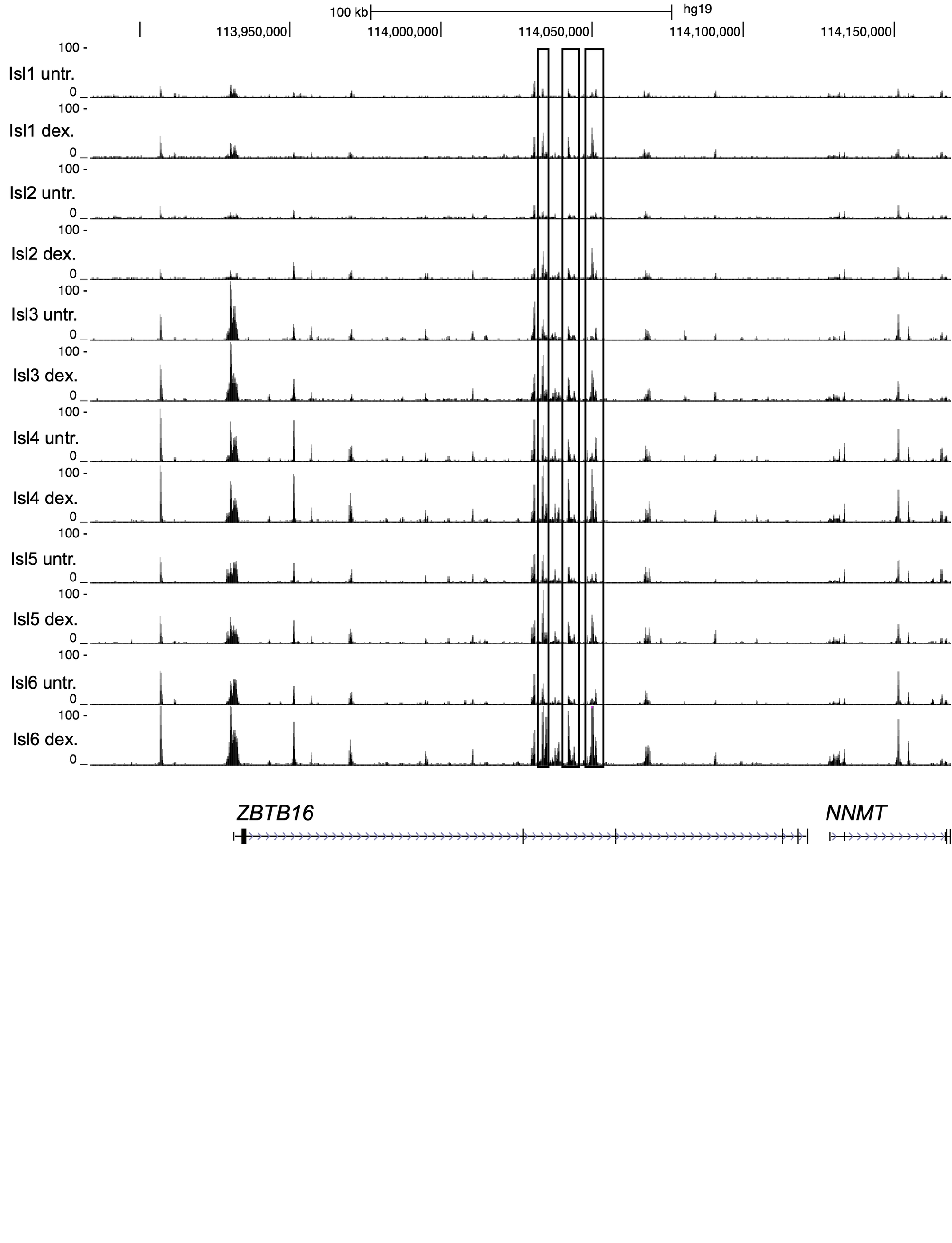

Supplement: S2 Fig — RPKM normalized ATAC-seq signal for individual islet sample in high-dose glucocorticoid treated and untreated islets. Sites with differences in chromatin accessibility across conditions are highlighted. (TIF) [file pgen.1009531.s002.tif]

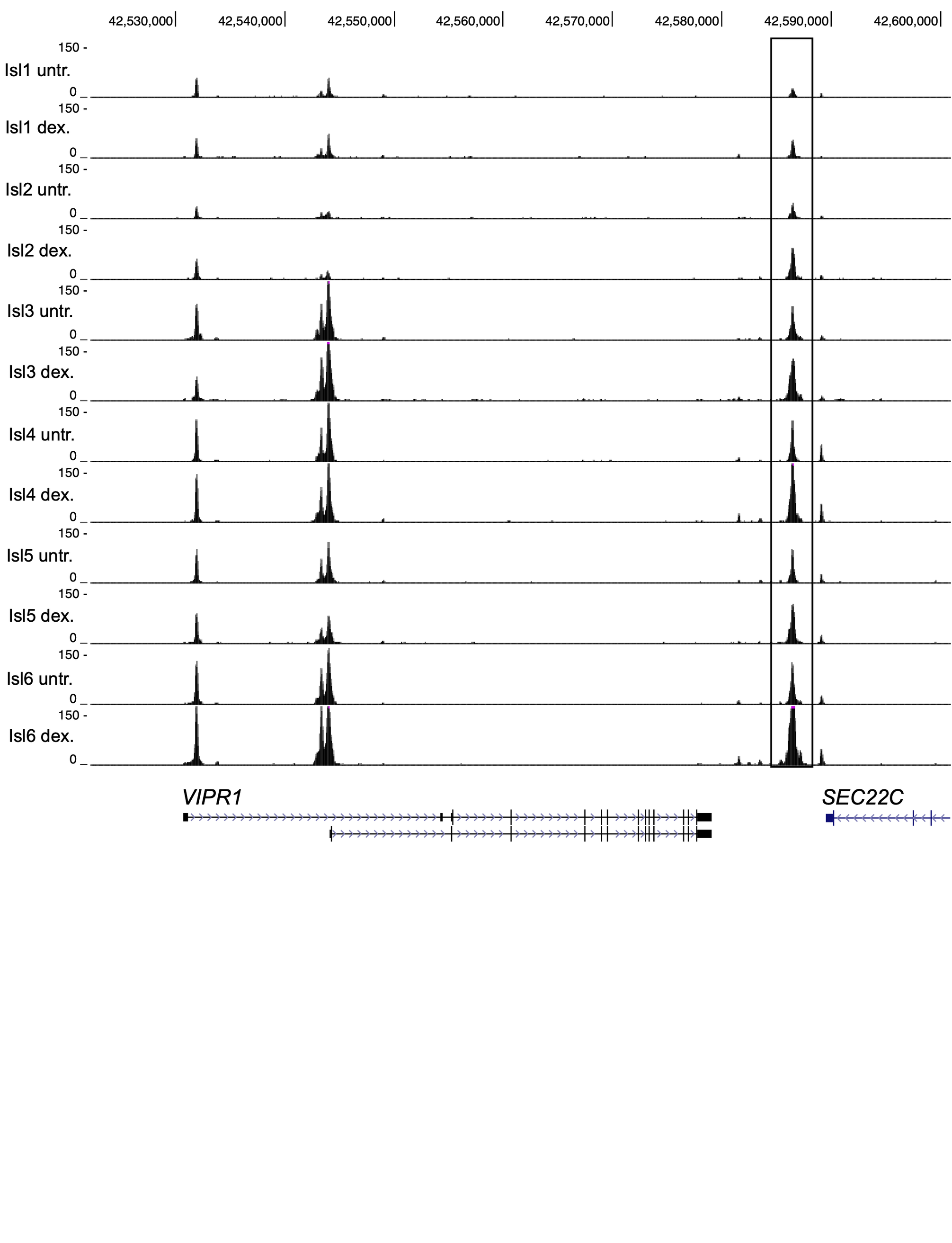

Supplement: S3 Fig — RPKM normalized ATAC-seq signal for individual islet sample in high-dose glucocorticoid treated and untreated islets. Sites with differences in chromatin accessibility across conditions are highlighted. (TIF) [file pgen.1009531.s003.tif]

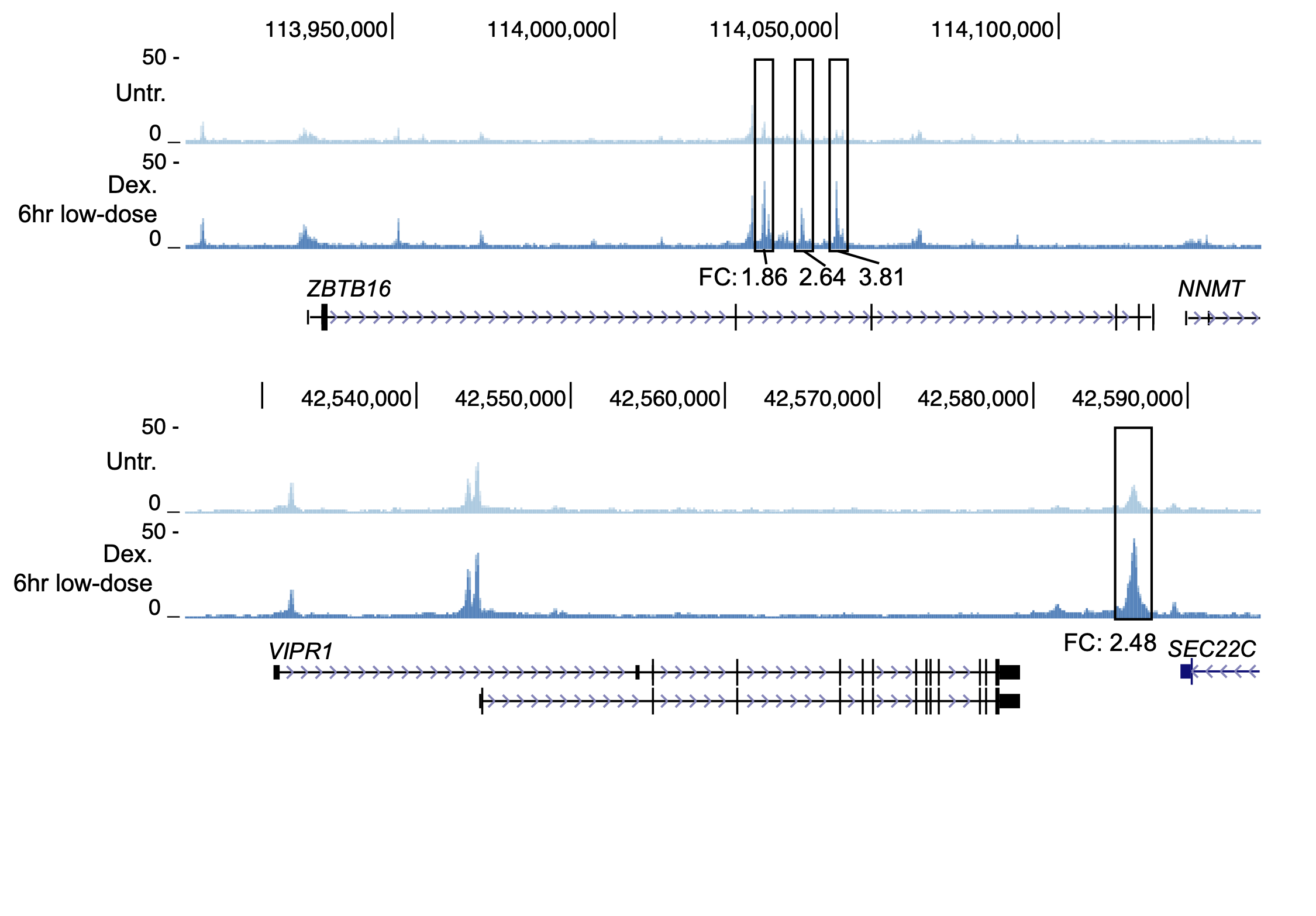

Supplement: S4 Fig — RPKM normalized ATAC-seq signal in low-dose (4ng/mL for 6hr) glucocorticoid treated and untreated islets at the (A) ZBTB16 and (B) VIPR1 loci. Sites induced by glucocorticoid treatment are highlighted. (TIF) [file pgen.1009531.s004.tif]

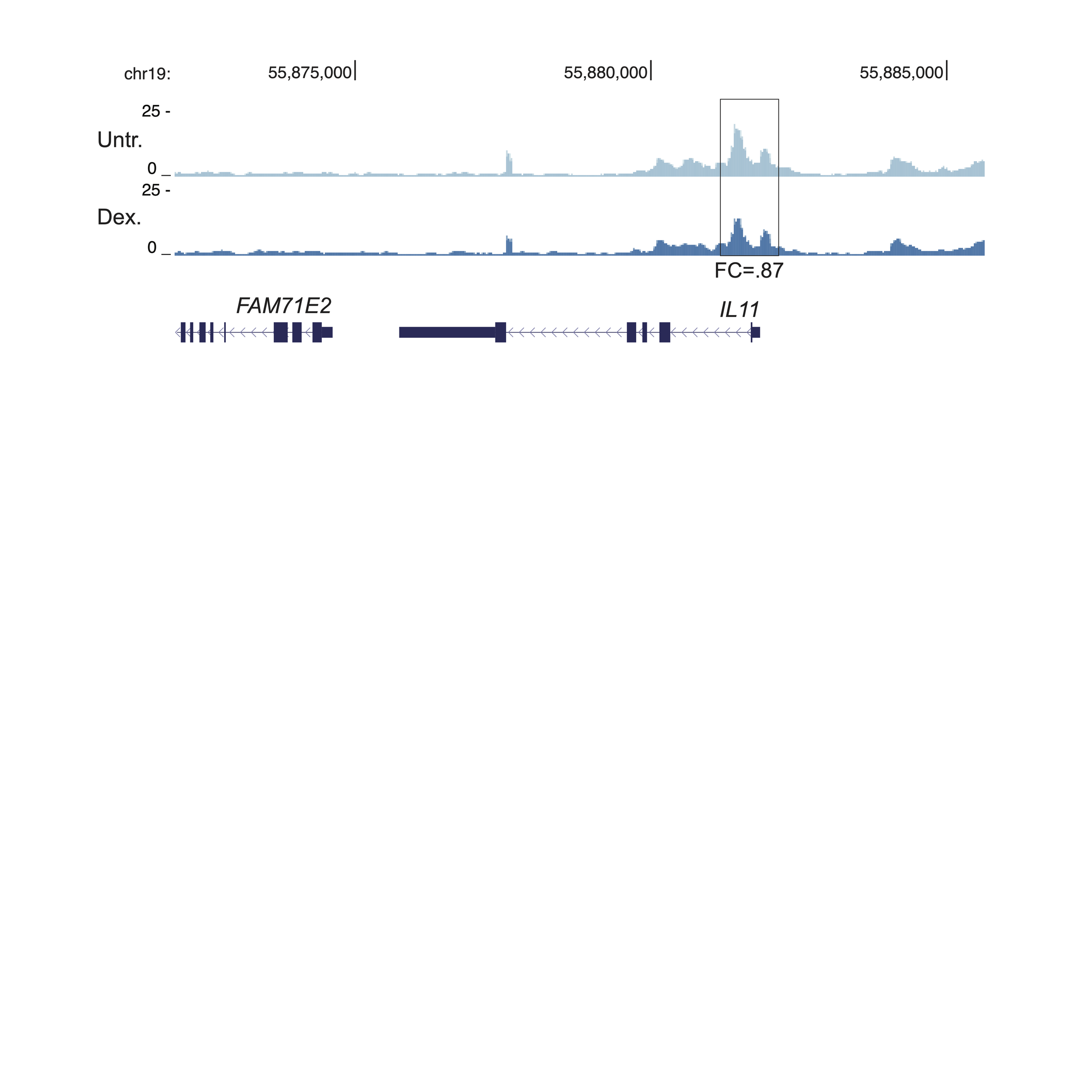

Supplement: S5 Fig — RPKM normalized ATAC-seq signal in high-dose glucocorticoid treated and untreated islets at the IL11 locus. The IL11 promoter which has reduced accessibility in glucocorticoid treated islets at high dose is highlighted. (TIF) [file pgen.1009531.s005.tif]

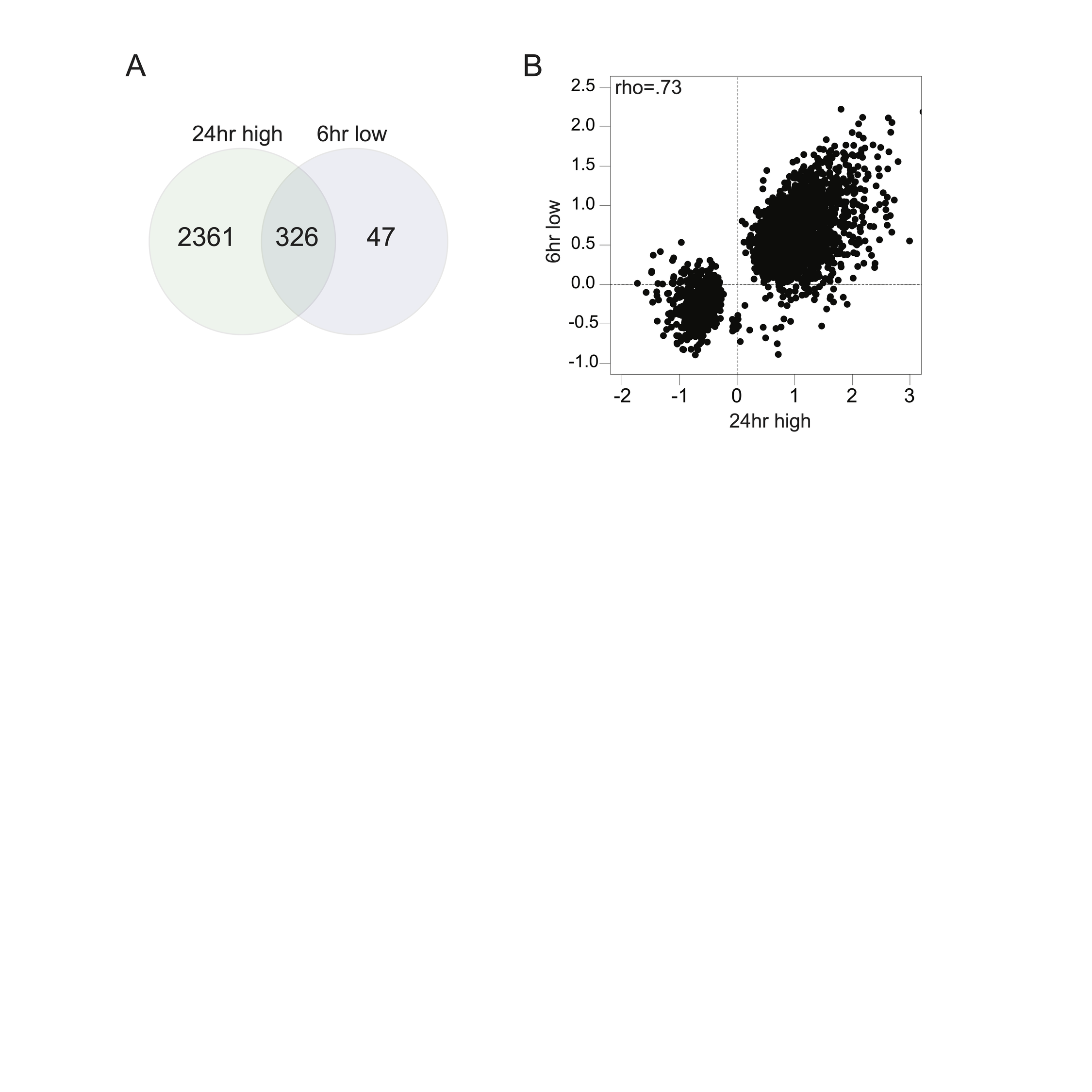

Supplement: S6 Fig — (A) Venn diagram of overlap in sites with differential activity in high-dose (100ng/mL for 24hr, n = 6) and low-dose (4ng/mL for 6hr, n = 3) glucocorticoid treatment. (B) Effects of high-dose and low-dose glucocorticoid treatment on sites with significant differential activity in either treatment. (TIF) [file pgen.1009531.s006.tif]

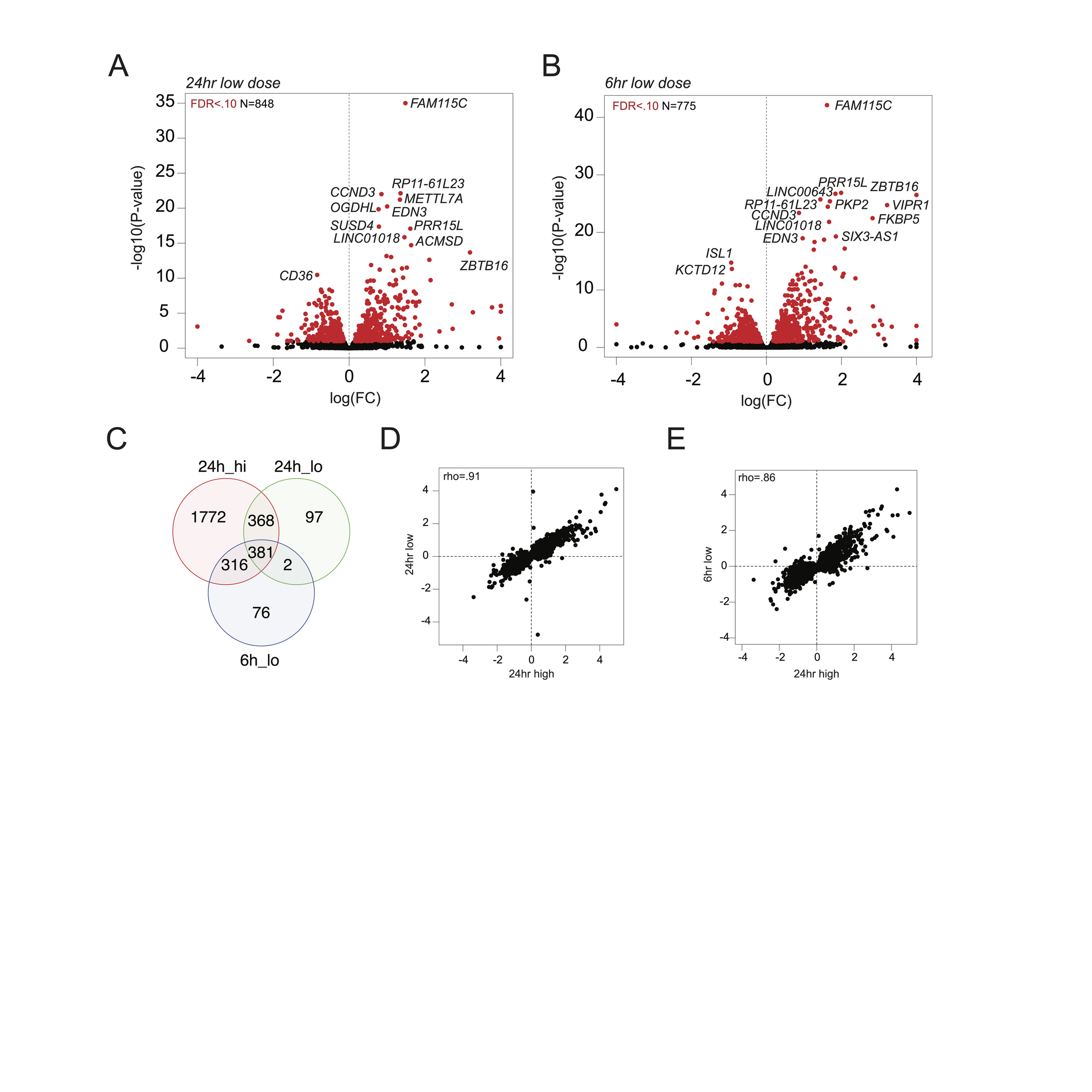

Supplement: S7 Fig — (A,B) Volcano plot of differential gene expression in glucocorticoid-treated islets at low dose for 24hr or 6hr compared to untreated islets. Genes with significant differential expression (FDR < .10) are highlighted in red, and genes with most pronounced changes in expression are listed. (C) Venn diagram of overlap between genes differentially expressed in 24hr high (n = 6), 24hr low (n = 3), 6hr low (n = 3) glucocorticoid treatment. (D) Effects of 24hr high- and low-dose treatment on genes with significant differential expression in either treatment. (E) Effects of 24hr high- and 6hr low-dose treatment on genes with significant differential expression in either treatment. (TIF) [file pgen.1009531.s007.tif]

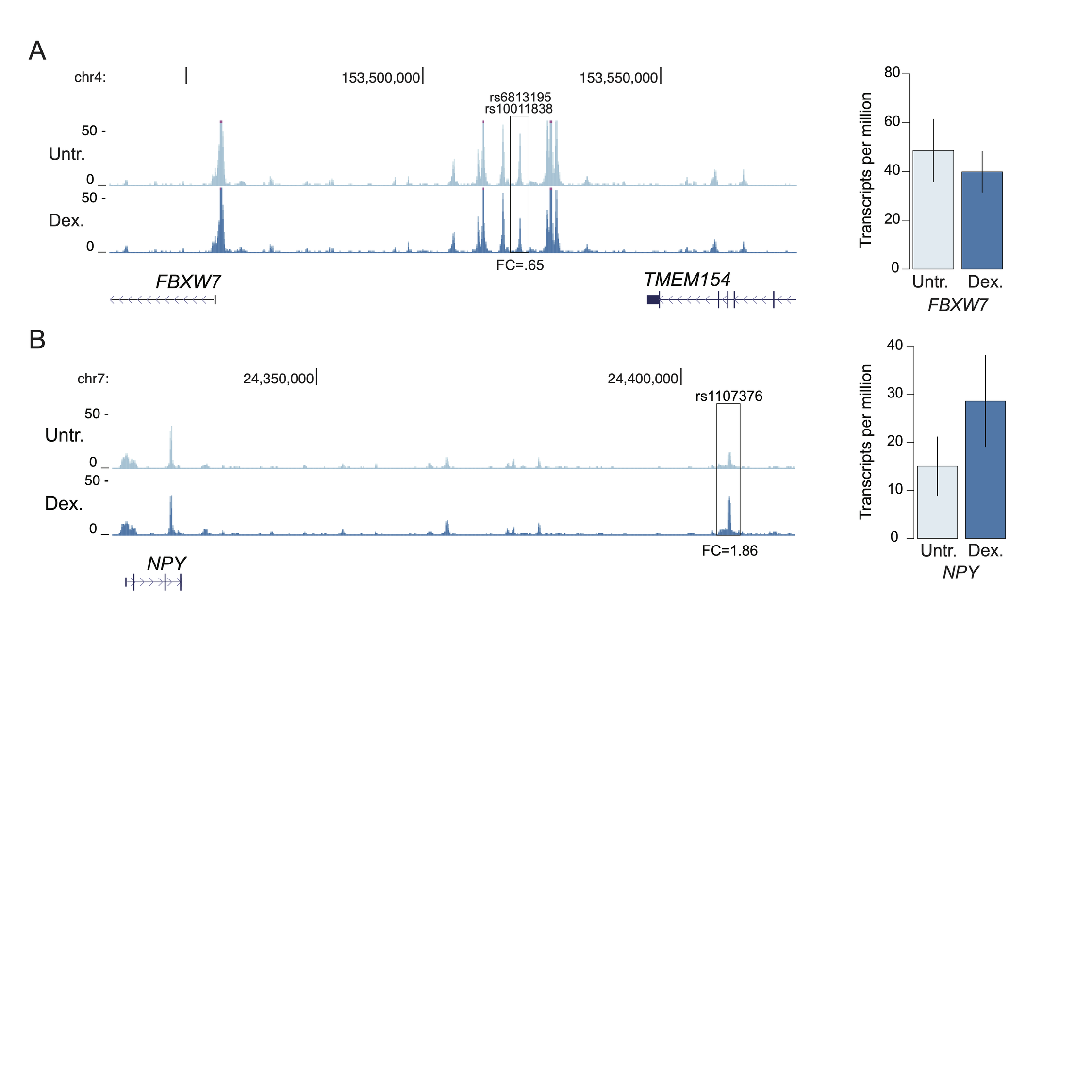

Supplement: S8 Fig — (A) Multiple variants at the FBXW7/TMEM154 locus mapped in a site with decreased activity and FBXW7 had decreased expression in glucocorticoid stimulation. (B) A variant at the NPY locus mapped in a site with increased activity and NPY had increased expression in glucocorticoid stimulation. Genome browser tracks represent RPKM normalized ATAC-seq signal, and expression bar plots represent mean expression and standard error. Values shown are from high-dose treatment. The fold-change (FC) in accessible chromatin signal in glucocorticoid treatment compared to untreated is indicated at highlighted sites. (TIF) [file pgen.1009531.s008.tif]
