# Supplementary material for: Glucocorticoid signaling in pancreatic islets modulates gene regulatory programs and genetic risk of type 2 diabetes
Source: PLoS Genet. 2021 May 13;17(5):e1009531. doi: 10.1371/journal.pgen.1009531 (PMC8183998; doi:10.1371/journal.pgen.1009531)
Supplement: Attachment — Submitted filename: Reviewer_response.docx [file pgen.1009531.s018.docx]

**We thank the reviewers for their helpful comments and in response we have made extensive revisions to the manuscript, which are detailed in-line.**

Reviewer 1

In this manuscript, Aylward et al identified how dexamethasone-induced glucocorticoid signaling in primary islet cells affect transcription by studying the changes in accessible chromatin and gene expression with ATACseq and RNAseq. First they discovered that ~3,000 genomic regions and ~1,000 genes responded to Dex treatment. The genomic regions were enriched for glucorticoid response elements and genes were enriched for steroid and lipid metabolism as well as ion transport. They then identified type 2 diabetes and glucose-associated genetic variants that overlap the Dex-responsive genomic regions and identified variants that had allelic imbalance in chromatin accessibility and had an effect on gene expression. Overall, this ia very nice study with interesting and well-presented findings. The conclusions are supported by the data and the results are nice interpreted. I have only a few minor suggestions that are mostly grammar errors, etc.

Line 181: remove "both"

**We amended this sentence to remove the word ‘both’.**

Add H to indicate where the panel H is in Figure 2.

**We mistakenly labeled this figure panel – in the revised manuscript we corrected the reference to Figure 2H to refer instead to Figure 2G.**

Line 308: "These results demonstrate that T2D and glucose level variants are enriched..." I do not know if the results show enrichment. It certainly shows presence but of all the T2D-associated SNPs, is there enrichment in the GR-responsive sites? You can either do an enrichment analysis or rephrase this claim.

**We did previously perform an enrichment test of T2D and glucose level variants for GR-responsive islet sites compared to a background of islet sites. We have however modified these analyses in the revised manuscript to use the fgwas method to test for enrichment. These results are included in the main text and in Figure 4A:**

**Line 307:** *“We therefore tested for enrichment of diabetes and fasting glycemia associated variants in glucocorticoid-responsive islet chromatin sites using fgwas^40^ (see Methods). We observed enrichment of variants influencing T2D risk and blood sugar (glucose) levels in chromatin sites with differential activity in both high- and low-dose glucocorticoid treatment (T2D high-dose ln(enrich)=3.71, low-dose ln(enrich)=4.23; blood sugar high-dose ln(enrich)=3.92, low-dose ln(enrich)=6.20) (Figure 4A). Conversely, we observed no evidence for enrichment of T1D risk variants (high-dose ln(enrich)=-28.0, low-dose ln(enrich)=-23.8) (Figure 4A).”*

**We have also clarified the end of the section:**

**Line 355: *“****These results reveal that variants associated with T2D and glucose level are enriched in glucocorticoid-responsive chromatin sites in islets, including variants such as rs12712928 at the SIX2/3 locus which directly interact with glucocorticoid signaling to affect islet regulation.”*

Fig 2 caption - Line 569: remove x, i think you mean 6 donors.

**We have made this change on line 642 (now 9 donors):**

*“Principal components plot showing ATAC-seq signal for high-dose (red) and low-dose (blue) glucocorticoid-treated islets and untreated (grey) islets from 9 total donors.”*

Fig 2 caption - Line 571: It is not clear what you mean by average values? Is is the average value of the loadings of the principal components?

**Yes and we have clarified this statement:**

**Line 643:** *“Dashed lines connect assays from the same sample, and box plots on each axis represent the distribution of principal components of samples for each condition.”*

In Fig 2G/H, it seems that rs684374 is a quadallelic SNP. Therefore, please identify which allele you consider to be the ref allele (G?) and which one you consider to be the alt allele (C?) on the figure.

**We have labeled the reference (G) and alternate (C) alleles in Figure 2G. These are the only two alleles that are commonly observed in populations for this variant.**

Fig 4e/f, please identify which allele is ref and which is alt on the figure.

**We have labeled the reference (G) and alternate (C) alleles in Figure 4E/F**

Reviewer 2

In this work, Aylward et al. investigate how glucocorticoid dexamethasone treatment affects chromatin accessibility and gene expression in cultured islet cells by performing RNA-seq and ATAC-seq. The authors identify differentially accessible sites and differentially expressed genes, enriched for pathways relevant to glucocorticoid signaling and inflammatory/stress response. The authors then use these profiles to fine map T2D GWAS loci. The study design is reasonable and the work could be quite useful to the community. I have the following comments:

To obtain the consensus set of ATAC-seq peaks across conditions and replicates, the authors merged peaks called in individual samples. Were sequencing depth differences in each replicate accounted for in this process? Eg. peaks could be identified in a particular condition owing to just higher depth, these would go into the final peak set and could be identified as differentially expressed?

**Sequencing depth was not considered in merging peaks across samples to define the consensus peak set. However, sequencing depth per sample is indeed accounted for in the statistical model of DESeq2 when identifying peaks with differential accessibility between conditions. This therefore precludes the possibility of peaks being called as differentially accessible simply due to differences in sequencing depth. In fact, DESeq2 has been demonstrated to have the best specificity to true positives of standard DAC methods (Gontarz et al Sci Rep 2020).**

**In order to provide confidence that the peaks in consensus set are reproducible across samples, in the revised manuscript we used IDR (Irreproducible Discovery Rate) developed by the ENCODE consortium to identify reproducible peaks. We applied IDR to replicate samples from each condition and considered peaks reproducible at FDR<.01. In total there were 127,228 reproducible peaks in this new consensus peak set, and we used these peaks for differential and other downstream analyses. These updated results are described in the main text and methods:**

**Line 157:** *“We first defined a canonical set of 127,228 islet accessible chromatin sites genome-wide by comparing replicate samples using IDR (see Methods, Supplemental Table 2).”*

**Line 468: *“****We first used Irreproducible Discovery Rate (IDR) to define a set of canonical ATAC-seq sites for differential analysis. In brief, for each condition separately, we pooled reads across all assays and randomly split the pooled reads into two ‘pseudo-replicates’. For the pooled and ‘pseudo-replicate’ data we called candidate peaks using MACS2^62^ with the parameters ‘—extsize 150 –keep-dup all –shift -75 –nomodel -p 0.01’. We applied IDR to the ‘pseudo-replicate’ candidate peak calls and obtained the number of peaks at an IDR threshold of .01. We then sorted and filtered the pooled candidate peak calls based on this number. Finally, we merged the resulting peaks across conditions, where if two peaks overlapped, we retained the more significant peak, and considered these canonical sites for downstream analyses.”*

Fig 2B shows the number of upregulated or downregulated accessible chromatin sites under dex vs untreated condition. A more informative figure could show for each peak tested, effect sizes/fold changes and p values to better appreciate the spread of the data. On similar lines of Fig 2C etc.

**Per the reviewer suggestion we have replaced the bar plot in Fig 2B with a volcano plot.**

It is confusing why examples for motifs enriched up or downregulated noted in the main text (lines 151-156) are not all labeled in Fig 2C. I then realized that the panels 2C and 2D are flipped in the main text vs figure.

**We have exchanged Figure 2C and 2D to the correct order.**

In the examples mentioned in lines 151-156, FOXA1 is noted to be enriched in upregulated peaks whereas FOXA2 is noted to be enriched in downregulated peaks. Checking the JASPAR motif database, the motifs for FOXA1 and 2 are largely similar, so it is unclear what to interpret from these results. What motif database was used for this? Maybe there should be a fold change threshold applied, since enrichment p values could just be driven by the motif PWM information content.

**We appreciate the reviewer highlighting this point. The motif database we used was included with HOMER (http://homer.ucsd.edu/homer/motif/motifDatabase.html), and we clarified this in the methods. The example for down-regulated sites should have also been listed as FOXA1, and this has been corrected in the text. Thus, the same FOXA1 motif is enriched in both up- and down-regulated sites. The fold-change of FOXA1 is similar in up-regulated peaks (1.34) and down-regulated peaks (1.39) compared to background. We note that the FOXA2 motif is also enriched in both up- and down-regulated sites (see Supplementary Table 4). Up- and down-regulated sites are both enriched for FOXA1 ChIP-seq sites, suggesting this pattern isn’t an artifact of sequence motif definition.**

**One interpretation of this result is that FOXA TFs mark sites that respond to signals (either up or down) but may not contribute directly to the changes in activity in response to these signals. This is consistent with the known role of FOXA TFs as pioneer factors that prime sites to enable binding of signal-dependent TFs, and we have added this point to the discussion:**

**Line 388: *“****We also observed enrichment of FOXA binding in sites both up- and down-regulated in glucocorticoid signaling, suggesting these TFs preferentially mark sites that respond to signal-dependent TF activity in islets in line with their known function as pioneer factors.”*

Differing from other places in the text, line 268 mentions P values in the -log10 scale. These values are barely significant and it’s hard to interpret/appreciate the enrichment. Looks like the background set of ATAC peaks were sampled from ATAC peaks across all experiments. A comparison between differentially accessible sites and unaltered sites could be more appropriate.

**We appreciate the reviewer suggestion and have revised the enrichment analysis to use a more standard method fgwas. The values now reported in the text and shown in the figure are the resulting enrichment values and 95% confidence intervals. The results are in line with the previous analysis in showing strong enrichment of T2D and glucose associated variants for glucocorticoid responsive sites. These results are included in the main text:**

**Line 309:** *“We observed enrichment of variants influencing T2D risk and blood sugar (glucose) levels in chromatin sites with differential activity in both high- and low-dose glucocorticoid treatment (T2D high-dose ln(enrich)=3.71, low-dose ln(enrich)=4.23; blood sugar high-dose ln(enrich)=3.92, low-dose ln(enrich)=6.20) (Figure 4A). Conversely, we observed no evidence for enrichment of T1D risk variants (high-dose ln(enrich)=-28.0, low-dose ln(enrich)=-23.8) (Figure 4A).”*

For fine-mapped variants in differentially accessible sites, the authors nominated target genes using proximity and differential gene expression. As later shown in the manuscript for one locus, how many of the 412 variants have evidence of association with gene expression from islet eQTL data?

**We identified 71 variants with at least nominal evidence of eQTL association to a predicted target gene. These results are included in Supplemental Table 8 and described in the main text:**

**Line 332:** *“At 71 T2D- or glucose-associated variants we further observed evidence for association with target gene expression (eQTL) in islets (Supplemental Table 8); for example, rs1107376 was an islet eQTL for NPY (P=2.2x10^-21^).”*

Minor comments:

Line 123 is referring to chromatin accessibility so the intended figure reference was liekly 2D,E instead of “2B,C”. Also, panels are mislabeled in in the Fig 1 legend (lines 560-566).

**The references to and labeling of these panels have been corrected.**

Fig 1B and C show ‘untr’ first and then ‘dex’. For consistency, in Figs D and E could also show ‘untr’ first and then ‘dex’. Also, track colors in Figs 1D and E could be made consistent with colors in Fig 1 B and C.

**We have exchanged the treatment order in those panels and colored the signal tracks throughout the manuscript accordingly.**

Fig 1E, seems that the highlighted peak in ‘dex’ goes higher than the range 100. The differences could be clearer if the y ranges are extended in these tracks.

**We have adjusted the y-axis RPKM limits for these tracks.**

Line 133 mentions that the PCA was performed on “read counts” which sounds like no normalization was performed. The methods section then notes that normalization was indeed performed. The main text should be clarified to avoid confusion.

**We have updated the phrasing to specify PCA was performed on normalized read counts (now line 148).**

In figures 1D, 1E and 2E, labeling a quantitative measure of the differences in the peak signals, i.e. fold changes from the differential analyses would be informative, on top of appreciating the differences qualitatively.

**We have labeled the peaks with their fold-change from the differential analyses in these figure panels as well as the relevant panels in the supplement.**

Figure 3D: what is the unit for distance on the x axis?

**The x-axis is “relative distance” as defined by the Bedtools utility reldist (https://bedtools.readthedocs.io/en/latest/content/tools/reldist.html) and is a dimensionless quantity. We have clarified this in the figure legend and methods:**

**Line 673: *“****Relative distance metric (from bedtools reldist) between accessible chromatin sites with differential activity (dex) and genes with differential expression compared to all chromatin sites (background).”*

**Line 529: *“****We calculated a relative distance metric with bedtools^72^ (reldist function) using either differential chromatin sites or a background of all islet accessible chromatin sites as the "a" argument and differentially expressed genes as the "b" argument.”*

Line 289 - it would be informative to mention the blood glucose GWAS SNP OR and P value. Presumably this is the lead variant as it gets the highest PPA? Line 294 mentions the Japan Biobank T2D P values, it would be useful to mention P values, OR and PPA from Diamante T2D data as well.

**We have updated the text to indicate that rs12712928 is indeed the lead variant for glucose association at the SIX3 locus in BBJ. We have included the effect (beta) and p-value for glucose association and T2D association in DIAMANTE and BBJ, as well as a reference to a large T2D GWAS in East Asians that was recently published (Spracklen et al Nature) where rs12712928 is the lead variant for the T2D signal. These results are described in the main text:**

**Line 336:** *“At the 2p21 locus associated with glucose level, lead variant rs12712928 (BBJ beta=.068, P=7.4x10^-46^) mapped in a chromatin site with increased activity in glucocorticoid signaling and was proximal to SIX2 and SIX3 which both had glucocorticoid-induced expression (Figure 4C,D, Supplemental Table 6, Supplemental Table 8). This variant had the highest posterior probability in fine-mapping data (PPA=.89), suggesting it is likely causal for glucose association at this locus. This variant also had evidence for T2D association in BBJ (beta=.048, P=2.1x10^-6^) and DIAMANTE beta=.022, P=.012), and was the lead variant at a T2D signal recently reported in East Asians (P=1.8x10^-14^)^42^.”*

Reviewer 3

The manuscript by Aylward and colleagues investigates gene expression and chromatin accessibility response to glucocorticoids in pancreatic islets. The manuscript is overall clear and logically builds through the different steps of analysis to demonstrate that 1. GC induce changes in gene expression and chromatin accessibility, 2. Which TFs are involved in this response, 3. That T2D risk variants are enriched in GC response chromatin accessibility regions and finally 4. Aims to provide experimental validation of GC-dependent allele-specific enhancer activity for a putative T2D causal variant. Overall the paper is based on solid data, however a few points require further clarification. Major points:

Allelic imbalance analysis. A binomial test is not appropriate for this analysis, as it does not account for over-dispersion. A beta-binomial distribution should be used.

**We have updated the allelic imbalance results to use beta-binomial tests. These results are now updated in the main text, methods and in Supplemental Table 5.**

**Line 205***: “Environmental stimuli can interact with genetic variation to affect chromatin accessibility and gene regulation. We therefore determined the effects of genetic variants on islet accessible chromatin in both glucocorticoid-treated and untreated conditions using allelic imbalance mapping. We performed microarray genotyping of islet samples and imputed genotypes into 39M variants (see Methods). For variants overlapping islet chromatin sites we obtained read counts in samples heterozygote for that variant, corrected for mapping bias using WASP and modeled the resulting counts for imbalance using a beta-binomial test. We then identified variants with at least nominal (P<.05) evidence for allelic imbalance in accessible chromatin from either glucocorticoid-treated or untreated islets (Supplemental Table 5). Among imbalanced variants, several mapped in glucocorticoid-responsive chromatin and had evidence for allelic effects in glucocorticoid-treated islets only, although we note that the effects were not significant after multiple test correction (FDR>.10). For example, variant rs684374 at 15q14 in a glucocorticoid-responsive site bound by GR had significant imbalance in glucocorticoid-treated islets only (GC ref frac.=.71, P=.015; untr. ref frac.=.58, P=.50) and was predicted to alter binding of GR (NR3C1) (Figure 2G). Similarly, variant rs11610384 at 12p11 in a glucocorticoid-responsive site bound by GR had significant imbalance in glucocorticoid-treated islets only (GC ref frac.=.80, P=1.3x10^-3^; untr. ref frac.=.45, P=.87) and disrupted nuclear receptor motifs (Supplemental Table 5).”*

**Line 547: *“****We identified heterozygous variant calls in each sample with read depth of at least 10 in both untreated and treated cells, and then used WASP^77^ to correct for reference mapping bias. We retained variants in each sample where both alleles were identified at least 3 times across untreated and treated cells. We then merged read counts at heterozygous SNPs from all samples in untreated and treated cells separately. We fit a beta-binomial model to the observed allele counts using the method of NPBin^78^. The parameters of the beta-binomial model were α=38.01 and β=36.62 with over-dispersion of .013 for untreated samples and α=40.78 and β=39.26 with over-dispersion of .012 for glucocorticoid-treated samples.* *We called imbalanced variants from the merged counts using a beta-binomial test, and then calculated q-values from the resulting beta-binomial p-values. We considered variants significant at an FDR<.10 and nominally significant at P<.05.”*

Please report GSEA results after multiple-test correction.

**We have updated both the main text in the paragraphs starting on lines 253 and 268 and Figure 3 to report q-values (FDR) instead of p-values, and Supplemental table 6 lists both p-values and q-values for enriched gene sets.**

Allele-specific enhancer activity at rs12712928. A formal test should be performed to compare the differential enhancer activity between the two alleles.

**A t-test was performed to determine differences in enhancer activity between variants alleles (Fig. 4E). We have clarified this in the main text:**

**Line 344:** *“The glucose increasing and T2D risk allele C had significantly reduced enhancer activity in both glucocorticoid-treated (T-test P=2.5x10^-6^) and untreated cells (T-test P=3.2x10^-4^) (Figure 4E).”*

**In the revised manuscript we have also added a permutation test of the difference between allelic effects in untreated compared to glucocorticoid treated islets (Fig. 4F) in the main text:**

**Line 346:** *“However, the allelic differences at this variant were more pronounced in glucocorticoid-treated cells (ref/alt ratio GC=6.85, 95% CI=3.4,10.2; untreated=1.78, 95% CI=1.23,2.32, permutation test P=5.1x10^-3^) (Figure 4F).”*

Differential chromatin accessibility analysis: How many peaks were tested for the differential chromatin accessibility analysis in DESEQ? Were the ATAC-seq data normalized prior to differential chromatin accessibility analysis? If they were not normalized, please repeat the analysis to reflect the same workflow used for differential gene expression.

**In the revised manuscript we modified the definition of peaks to include those reproducible across replicate samples defined using Irreproducible Discovery Rate (IDR) developed by the ENCODE consortium and identified 127,228 reproducible sites. These 127,288 sites were tested for differential accessibility using DESeq. We have included this number in the main text:**

**Line 157:** *“We first defined a canonical set of 127,228 islet accessible chromatin sites genome-wide by comparing replicate samples using IDR (see Methods, Supplemental Table 2). Among these canonical sites, there were 2,688 sites with significant evidence (FDR<.10) for differential activity in glucocorticoid signaling at high-dose treatment (Figure 2B, Supplemental Table 3).”*

**With respect to normalization we note that raw read counts are the proper input to DESeq2 for both ATAC-seq and RNA-seq analysis. Normalization is handled as an internal step of DESeq2 analysis (see Love et al Genome Biology). We have clarified the methods for the gene expression analysis:**

**Line 518: *“****To identify differentially expressed genes between treated and untreated samples we obtained raw expression counts from RSEM^72^ for the 20,480 genes and applied DESeq2^64^ with default settings including donor as a cofactor to model paired samples.”*

Minor points:

Line 171, please specify what type of cells are MIN6.

**We have specified that MIN6 are mouse insulinoma cells:**

**Line 198:** *“We tested the glucocorticoid-induced site at this locus (high-dose fold-change=1.78; P=1.0x10^-5^; low-dose fold-change=2.19; P=4.4x10^-4^) for enhancer activity in luciferase gene reporter assays in dexamethasone-treated and untreated MIN6 mouse insulinoma cells.”*

Figure 3E. Please include a legend to interpret the dot size. What is the scale of the X axis and why all dots align in the same position?

**As the dot plots were confusing to interpret, in the revised manuscript we have changed these figure panels to bar plots.**

Please report in the text the enrichment for T2D and glucose variants in GC-responsive chromatin.

**We have included enrichment values (note that the enrichment method has changed from the previous version) for diabetes and glucose association in the main text and Figure 4A:**

**Line 309:** *“We observed enrichment of variants influencing T2D risk and blood sugar (glucose) levels in chromatin sites with differential activity in both high- and low-dose glucocorticoid treatment (T2D high-dose ln(enrich)=3.71, low-dose ln(enrich)=4.23; blood sugar high-dose ln(enrich)=3.92, low-dose ln(enrich)=6.20) (Figure 4A). Conversely, we observed no evidence for enrichment of T1D risk variants (high-dose ln(enrich)=-28.0, low-dose ln(enrich)=-23.8) (Figure 4A).”*

What concentration of dexamethasone was used in the reporter gene assays? Please add this information in the methods.

**We clarified the concentration of dexamethasone used in reporter assays in the methods:**

**Line 603: *“****For stimulation 100 ng/mL Dexamethasone (Sigma) was added to the culture media.”*

Reviewer 4

Aylward and colleagues investigate the role of glucocorticoid signaling in pancreatic islets, using several high throughput sequencing analyses in human islet specimens, combined with analyses of publicly available datasets in order to determine causal regulatory profiles between GR signaling and T2D. Numerous steroid responsive genes are identified and confirmed after in vitro culture with dexamethasone. The authors identify genetic variants for glucose control and T2D risk overlaps with numerous corticosteroid responsive chromatin sites and focused on a highly specific causal link between diabetes risk and glucocorticoid signaling, driven by the key human beta cell genes SIX2 and SIX3. The authors conclude a role for glucocorticoid signaling in the genetic risk for T2D. General comments: The authors utilize multiple sophisticated sequencing and bioinformatics approaches to develop a regulatory map of glucocorticoid-responsive genes/chromatin in human pancreatic islets. Given the connections between Cushing’s syndrome and diabetes in humans, these results provide new results not previously available in human islets that may have bearing on both diseases. The study is well written and bioinformatics studies are thorough. However, there are several significant concerns, detailed below, with the experimental design of the study on a relative small number of islet samples could lead to conclusions that may not truly approximate the role of glucocorticoid signaling in beta cells and may lead to overinterpretation of the role of GR in the development of T2D. Specific Comments:

A major concern is the use of dexamethasone at an exceedingly high dose of 100 ng/mL for 24 hours to profile glucocorticoid signaling. This dose is 25-fold higher than physiologic glucocorticoid doses and also dramatically higher than what are even observed in patients with Cushing’s syndrome. At this dose, it is highly likely that the results will lead the authors to interpret roles for glucocorticoids that would not reflect physiologic changes related to T2D in vivo. It is crucial for the authors to perform their studies at (1) physiologic glucocorticoid doses, (2) a dose response curve favoring lower glucocorticoid doses, or (3) in the presence of pharmacologic inhibitors to ensure their results are specific and not due to off target effects.

**We appreciate the reviewer comment and in response have included additional genomic data using lower doses of glucocorticoid treatment in the revised manuscript. We generated RNA-seq from 3x islets treated with 4ng/mL dexamethasone for 6hr and 4ng/mL dexamethasone for 24hr (n=3) as well as untreated. We also generated RNA-seq from 3x additional islets treated with 100ng/mL for 24hr to supplement the data included in the previous version of the manuscript (total n=6). We further generated ATAC-seq from 3x islets treated with 4ng/mL dexamethasone for 6hr (n=3) as well as untreated. We have made substantial revisions throughout the entire manuscript in incorporating the analysis of these data.**

**At the dexamethasone-responsive genes *ZBTB16, VIPR1* and *IL11* highlighted in Figure 1 we observed induction of gene expression and chromatin accessibility in low dose treatment as seen with high dose treatment in the original manuscript. These results are described in the main text on lines 124, 131 and 138 and included in Supplemental Figure 1 and 4.**

**Line 124:** *“In order to determine the effects of glucocorticoid signaling on pancreatic islet regulation, we cultured primary islet cells in vitro with dexamethasone at several different doses (100 ng/mL for 24hr, 4 ng/mL for 6hr and 24hr) as well as in untreated conditions and measured accessible chromatin and gene expression levels in both treated and untreated cells.”*

**Line 131:** *“Across replicate samples we observed changes in expression levels of genes both known to be induced by dexamethasone such as ZBTB16^35–37^ and VIPR1^38^ as well as those suppressed by dexamethasone such as IL11^39^ in both the high-dose (100 ng/mL) and low-dose (4 ng/mL) treatments (Figure 1B, Figure 1C, Supplemental Figure 1A-C).”*

**Line 138:** *“For example, accessible chromatin signal was notably induced at several sites proximal to the ZBTB16 and VIPR1 genes in dexamethasone-treated compared to untreated islets in both high- and low-dose treatments (Figure 1D,E, Supplemental Figure 2, Supplemental Figure 3, Supplemental Figure 4).”*

**For ATAC-seq data in principal components analysis the effects of low-dose treatment were intermediate to high-dose treatment, demonstrating that the low-dose treatment has a more subtle effect on the same regions of accessible chromatin. These results are shown in Figure 2A and described in the main text on line 151. Among chromatin sites with differential activity, we observed strong concordance in the effects of low- and high-dose treatment. These results are described in the main text on line 167 and included in Supplemental Table 3 and Supplemental Figure 6. Furthermore, sites with significant differential activity in low-dose treatment had highly similar TF enrichments including GR and other nuclear hormone receptor sequence motifs and ChIP-seq binding sites, which is described in the main text on line 175 and 184 and included in Supplemental Table 4.**

**Line 151:** *“We observed reproducible differences in accessible chromatin profiles in dexamethasone-treated compared to untreated islets across replicate samples, where the effects of low-dose treatment (4 ng/mL, n=3) were intermediate to high-dose treatment (100 ng/mL, n=6) relative to untreated samples (n=9) (Figure 2A).”*

**Line 167:** *“At low-dose treatment, 373 sites had differential activity (FDR<.10) in glucocorticoid signaling, where the majority (350) were up-regulated (Supplemental Table 3). Among sites with differential activity in either treatment, the effects in high- and low-dose were highly concordant (Spearman ρ=.72, P<2.2x10^-16^) (Supplemental Figure 6A,B).”*

**Line 175:** *“The most enriched sequence motifs in up-regulated sites for both high- and low-dose treatment were for glucocorticoid and other steroid hormone response elements (high-dose: GRE P=1x10^-340^, ARE P=1x10^-302^, PGR P=1x10^-280^; low-dose: GRE P=1x10^-73^, ARE P=1x10^-66^, PGR P=1x10^-62^), in addition to lesser enrichment for TFs relevant to islet function (FOXA1: high-dose P=1x10^-5^, low-dose P=1x10^-3^) (Figure 2C, Supplemental Table 4).”*

**Line 184:** *“We observed strongest enrichment of up-regulated accessible chromatin sites in both high- and low-dose treatment for glucocorticoid receptor (NR3C1) binding sites (high-dose ratio=3.7, P=1.7x10^-294^, low-dose ratio=5.4, P=2.1x10^-129^), and less pronounced enrichment for binding sites of FOXA1 (high-dose ratio=1.7, P=1.6x10^-55^; low-dose ratio=2.3, P=2.3x10^-30^) and other TFs (Figure 2D, Supplemental Table 4).”*

**For RNA-seq data, similar to ATAC-seq, in principal components analysis the effects of low-dose treatment on gene expression were intermediate to the high-dose treatment demonstrating that low-dose treatment has a more subtle effect on the expression of the same genes. These results are described in the main text on line 233 and shown in Figure 3A. Among genes with differential expression, we observed strong concordance in the effects of low- and high-dose treatment. These results are described in the main text on line 248 and included in Supplemental Figure 7 and Supplemental Table 6. Genes with the most significant differential expression were highly similar across treatments and the top genes are highlighted for the low-dose treatments in Supplemental Figure 7. Furthermore, genes with differential expression in low-dose treatment were enriched for similar cellular processes as in the high-dose treatment, which is described in the main text on lines 276 and 289 and included in Supplemental Table 7.**

**Line 233:** *“There were again reproducible differences in expression levels across replicate samples, where the effects of low-dose treatment (4 ng/mL at 24hr, n=3; 4 ng/mL at 6hr, n=3) were intermediate to high-dose treatment (100 ng/mL at 24hr, n=6) relative to untreated samples (n=6) (Figure 3A).”*

**Line 248:** *“At low-dose treatment 775 and 848 genes had differential expression (FDR<.10) at 6hr and 24hr, respectively (Supplemental Table 6, Supplemental Figure 7A,B). Among genes differentially expressed in either treatment, the effects in high- and low-dose were highly concordant (24hr low-dose ρ=.91, P<2.2x10^-16^; 6hr low-dose ρ=.86, P<2.2x10^-16^) (Supplemental Figure 7C-E).”*

**Line 276:** *“Similar gene sets were enriched among genes up-regulated in low-dose treatments (Supplemental Table 7).”*

**Line 289:** *“Similar gene sets were enriched among genes down-regulated in low-dose treatments (Supplemental Table 7).”*

**Finally, T2D- and glucose-associated variants were strongly enriched in chromatin sites induced by lower dose dexamethasone treatment. These results are described in the main text on line 309 and 323 and included in Figure 4A. For example, both of the sites highlighted in Figure 4 at the *SCD5* and *SIX2/3* loci that harbor glucose and T2D associated variants are also significantly induced in lower dose treatment.**

**Line 309***: “We observed enrichment of variants influencing T2D risk and blood sugar (glucose) levels in chromatin sites with differential activity in both high- and low-dose glucocorticoid treatment (T2D high-dose ln(enrich)=3.71, low-dose ln(enrich)=4.23; blood sugar high-dose ln(enrich)=3.92, low-dose ln(enrich)=6.20) (Figure 4A). Conversely, we observed no evidence for enrichment of T1D risk variants (high-dose ln(enrich)=-28.0, low-dose ln(enrich)=-23.8) (Figure 4A).”*

**Line 323:** *“For example, T2D-associated variants at the 11q12 locus mapped in a site induced by high- and low-dose glucocorticoids proximal to SCD5 and TMEM150C which both had up-regulated expression (Figure 4B, Supplemental Table 3, Supplemental Table 8).”*

**Together these results demonstrate broad consistency in the effects of a lower dose (4ng/mL) of dexamethasone on the islet genomic profiles reported in the initial submission using a higher dose and confirm the relevance of our findings to islet physiology and diabetes risk.**

While the authors did confirm the activation of GR responsive genes, they also note numerous additional nuclear hormone receptors activated by dexamethasone (given enrichment for ARE and PGR sites). These effects are again likely related to supraphysiologic glucocorticoid levels. It would be crucial to determine if these sites are enriched with more appropriate glucocorticoid doses. Alternatively, these sites may represent a nexus of overlapping binding sites shared for glucocorticoid and other steroid hormones. Thus, the authors should consider treating islets with ligands for these additional steroid responsive hormone receptors to see if GR elements are similarly induced.

**As described in response to the comment above, we generated ATAC-seq data from islets treated with lower dose (4ng/mL) dexamethasone treated as well as untreated. Sites with differential activity in low-dose treatment were most enriched for GR as well as AR and PGR binding motifs, demonstrating that the enrichment for these other motifs was not due to the higher dose of dexamethasone. These results are described in the main text on line 175 and included in Supplemental Table 4:**

**Line 175:** *“The most enriched sequence motifs in up-regulated sites for both high- and low-dose treatment were for glucocorticoid and other steroid hormone response elements (high-dose: GRE P=1x10^-340^, ARE P=1x10^-302^, PGR P=1x10^-280^; low-dose: GRE P=1x10^-73^, ARE P=1x10^-66^, PGR P=1x10^-62^), in addition to lesser enrichment for TFs relevant to islet function (FOXA1: high-dose P=1x10^-5^, low-dose P=1x10^-3^) (Figure 2C, Supplemental Table 4).”*

**The likely reason for the observed enrichment of motifs for other nuclear hormone receptors is that the binding motifs are all very similar (see Figure R1 below). Determining whether the same sites are actually bound by these other receptors is outside the scope of this study, but we included additional discussion to address this on line 406:**

**Line 406: *“****Given the similarity in binding motifs of many nuclear hormone receptors and the enrichment of glucocorticoid responsive sites for androgen and progesterone receptor motifs, the effects of GR on islet gene regulation may overlap with other nuclear receptors by acting on shared chromatin sites^58^.”*

**Figure R1:** Sequence logos of GRE, ARE and PGR motifs included in the HOMER database

Several of the results are confusing as it is unclear if the authors infer whether glucocorticoid activity is favorable as “signaling increases the activity of genes involved in islet function and insulin secretion while suppressing inflammatory and proliferative gene activity”, or whether it is detrimental due to impairment of key ion channels. At the doses applied, it would be expected that beta cell function would be impaired as key beta cells genes including NKX6.1 are downregulated.

**We appreciate the reviewer comment and as key beta cell genes are downregulated it is likely that beta cell function is impaired particularly at high-dose treatment, but we observe similar patterns in the low-dose treatments as well. We have modified the text and discussion to reflect this:**

**Line 291: *“****Down-regulated genes included those involved in the inflammatory response such as IL6, STAT5B, STAT3, STAT4, SMAD3, CXCL12, CCL2, CD44, CD36, RELB, IRF1, extracellular matrix formation such matrix metalloproteinase genes such as MMP3, MMP7, MMP9 and matrix components such as LAMA4 and LAMC2, islet function and pancreatic differentiation such as ISL1, PAX6, NKX6-1, HES1 and JAG1, and proliferation and growth factors such as PDGFA, PDGFB, FGF2, TGFB3 and VEGFA (Figure 3F, Supplemental Table 6).”*

**Line 298:** *“These results demonstrate that glucocorticoid signaling in islets up-regulates genes involved in steroid and lipid metabolism and ion channel activity, and down-regulates key genes in islet function as well as genes involved in inflammation, proliferation and extracellular matrix formation.”*

**Line 383:** *“Conversely, glucocorticoid signaling down-regulated inflammatory programs, in line with previous reports and the known function of glucocorticoids^2,17,48^, as well as key genes involved in islet function such as NKX6-1, PAX6, RFX6, and ISL1.”*

Minor Comments:

The legend for Figure 1 is mislabeled as Figure 1E is missing but 1C is listed twice.

**We corrected the legend for Figure 1.**

What do error bars represent in their figures? This is not clear. SD, SEM?

**We have defined error bars in figure captions where missing.**
