# Supplementary material for: Glucocorticoid signaling in pancreatic islets modulates gene regulatory programs and genetic risk of type 2 diabetes
Source: PLoS Genet. 2021 May 13;17(5):e1009531. doi: 10.1371/journal.pgen.1009531 (PMC8183998; doi:10.1371/journal.pgen.1009531)
Supplement: Attachment — Submitted filename: Reviewer_response.docx [file pgen.1009531.s019.docx]

**We thank the reviewers for their additional comments, and responses to these comments are provided in-line.**

Reviewer #1: I had minor comments for the original submission. The authors satisfactorily addressed them.

Reviewer #2: The authors have made numerous edits and included additional experiments and analyses that all helped address my comments. I also find responses to other reviewer comments reasonable as well. These modifications have made the manuscript more robust. As promised, the authors should make sure to share all raw and processed data through appropriate channels.

**We have deposited the raw data in GEO under accession number GSE167250.**

Reviewer #3: The revised version of this manuscript addresses all my previous comments. The authors have done a great job in responding to the comments of all reviewers.

I only have two additional comments on the new and corrected analysis of allelic imbalance that is presented in this revised version. The results reported in the paragraph starting at line 205 should focus on sites with significant imbalance after multiple test correction, e.g. q value < 0.1 (equivalent to 10% FDR). The manuscript is currently using a nominally significant p-value threshold of 0.05. Additionally, if any inference is to be presented on condition-specific allelic imbalance, a formal test should be performed to contrast the allelic imbalance in the treatment and control conditions.

**We have modified the results to report only variants with allelic imbalance that are significant at FDR<.10. These changes are included in the main text on line 205, the methods on line 545, Supplemental Table 5 and Figure 2.**

**We have performed an additional analysis using a proportions test to identify variants with significant difference in allelic effects between treated and control cells. These changes are included in the main text on line 213, in the methods on line 557 and in Supplemental Table 5 and Figure 2.**

Reviewer #4: Authors have addressed my remaining concerns with the manuscript.
